# Phagocytosis-Regulators-Based Signature to Predict the Prognosis and Chemotherapy Resistance for Breast Cancer Patients

**DOI:** 10.3390/ijms231810312

**Published:** 2022-09-07

**Authors:** Juan Feng, Jun Ren, Xiuqi Li, Xue Zhang, Qingfeng Yang, Zankai Wu, Le Cui, Lingxia Liao, Yiping Gong, Dedong Cao

**Affiliations:** 1 Department of Breast and Thyroid Surgery, Renmin Hospital of Wuhan University, Wuhan 430060, China; 2 Department of Gastrointestinal Surgery II, Renmin Hospital of Wuhan University, Wuhan 430060, China; 3 Department of Rehabilitation, Renmin Hospital of Wuhan University, Wuhan 430060, China; 4 Department of Oncology, Renmin Hospital of Wuhan University, Wuhan 430060, China

**Keywords:** breast cancer, phagocytosis, survival, drug resistance, biomarker

## Abstract

Phagocytosis is crucial in tumor surveillance and immune function. The association between phagocytosis and the outcomes of breast cancer patients has not been well-determined. In this study, data were downloaded from the cancer genome atlas (TCGA) and gene expression omnibus (GEO) databases to investigate the role of phagocytosis in breast cancer. Data from the TCGA and GEO databases were used to investigate the prognostic role of phagocytosis in breast cancer. Then, we performed pathway enrichment analysis, copy number variation (CNV) and single-nucleotide variant (SNV) analyses, immune infiltration analysis, calculation of tumor purity, stromal score, and immune score, and consistent clustering. We also constructed a phagocytosis-regulators-based signature system to examine its association in survival and drug response. The genomic and expression differences in the phagocytosis regulators in breast cancer were systematically analyzed, explaining the widespread dysregulation of phagocytosis regulators. Using the investigated association of phagocytosis regulators with the prognosis and tumor immune environment, we constructed a prognostic model based on phagocytosis regulators. We discovered that patients with high risk scores had a poor prognosis and were negatively associated with immune functions. The model had preferential predictive performance and significantly consistent drug-resistance prediction results. Our findings suggest that the phagocytosis-factors-based scoring system can be used as a novel prognostic factor, serving as a powerful reference tool for predicting prognosis and developing methods against drug resistance.

## 1. Introduction

In the past ten years, the research on the heterogeneity of breast cancer has made great progress, and a series of treatment methods has been proposed for breast cancer with different molecular phenotypes [1].The classification of luminal A (hormone receptor positive (HR+)/human epidermal growth factor receptor-2 (HER2)−, and low levels of protein Ki-67), luminal B (ER+, progesterone receptor (PR)+/−,HER2−), luminal B-like(ER+, PR+/−,HER2+), HER2-enriched (HR−/HER2+), and triple-negative breast cancer(TNBC) (HR−/HER2−) has entered clinical guideline [2]. The different classification methods of breast cancer are unsuitable for all treatment strategies. Therefore, more novel classification methods have been proposed, but consensus has not yet been reached.

At the same time, the emergence of immunotherapy, especially immune checkpoint inhibitors, has provided a new way of thinking for treating solid tumors [3]. Although the clinical application of this therapy can bring significant curative effects to tumor patients and improve survival, a considerable number of patients still fail to benefit from immunotherapy for some reason [4]. Moreover, there are currently no effective methods for the application and efficacy evaluation of immunotherapy [5]. Therefore, improving the efficacy, reducing the threshold, and evaluating the timely efficacy and response of immunotherapy remain significant issues.

The phagocytosis of macrophages is an important part of the immune regulation of the immune system, and the severe dysfunction of macrophages also participates in the occurrence and development of tumors. Here, by analyzing the phagocytosis regulators of phagocytes, we constructed a signature that can predict patient prognosis and immunotherapy efficacy, and we used unsupervised machine learning methods to verify the accuracy and application value of this signature.

## 2. Results

The process of this study is presented in Supplementary Figure S1.

### 2.1. Phagocytosis Regulators

Here, we extracted 29 phagocytosis factors from two articles of PMID: 34497417 and PMID: 30397336 [6,7]. In addition, we continued to select genes of two terms, GOBP-REGULATION OF PHAGOCYTOSIS and G OBP MACROPHAGE ACTIVATION from MSIGDB, as phagocytosis regulators. A total of 214 genes were obtained from the above four sources (Supplementary Table S1).

### 2.2. Phagocytosis Regulators Regulate the Involvement of Macrophages in the Occurrence and Development of Breast Cancer

**Figure 1:**
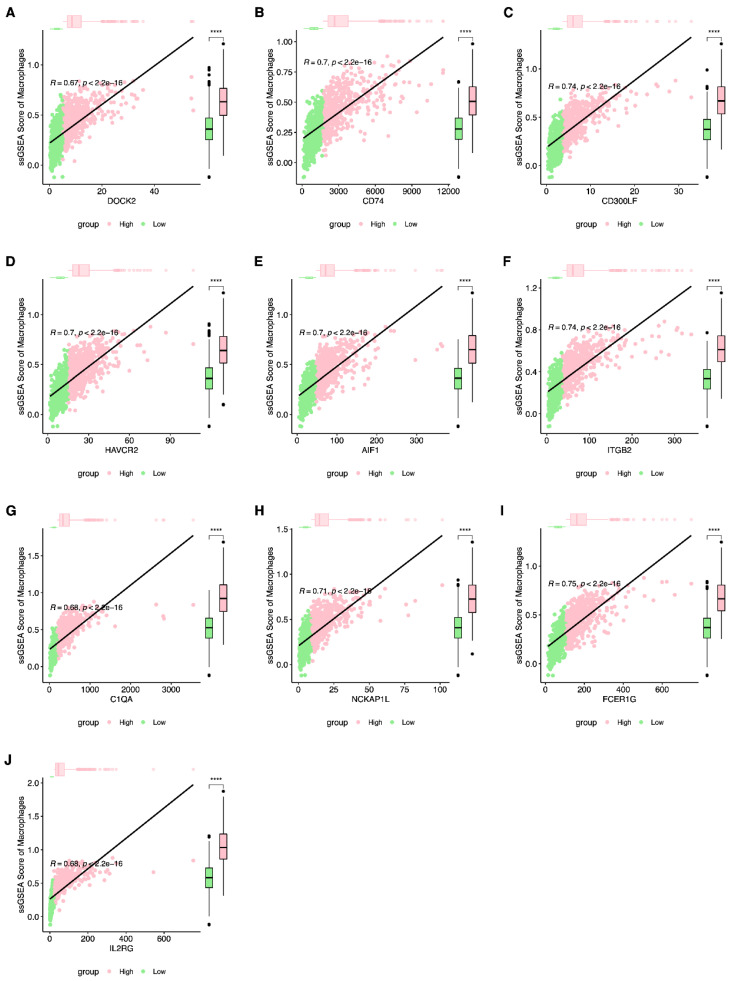
The correlation of expression of macrophage and macrophage enrichment score with top 10 phagocytic factors. (**A**) *DOCK2*; (**B**) *CD74*; (**C**) *CD300LF*; (**D**) *HAVCR2*; (**E**) *AIF1*; (**F**) *ITGB2*; (**G**) *C1QA*; (**H**) *NCKAP1L*; (**I**) *FCER1G*; (**J**) *IL2RG*. (**** *p* < 0.0001).

First, we focused on phagocytosis regulators that regulate macrophage phagocytosis. Using the immune cell signature genes in the literature PMID28052254, we extracted macrophage signature genes [8]. Based on the macrophage gene set, we applied ssGSEA to analyze the enrichment of TCGA-BRCA samples on the macrophage gene set. After obtaining the macrophage enrichment score, using Pearson correlation analysis, we calculated the correlation between phagocytosis factors and the macrophage enrichment score. At the same time, the samples were grouped with high and low expression levels based on the median expression of phagocytic factors, and the differences in enrichment scores between high- and low-expression groups were counted. Among them, there were 28 genes with a correlation coefficient>0.5 and *p*-value <0.001 (Table 1, Figure 1 and Figure S2).

**Figure 2:**
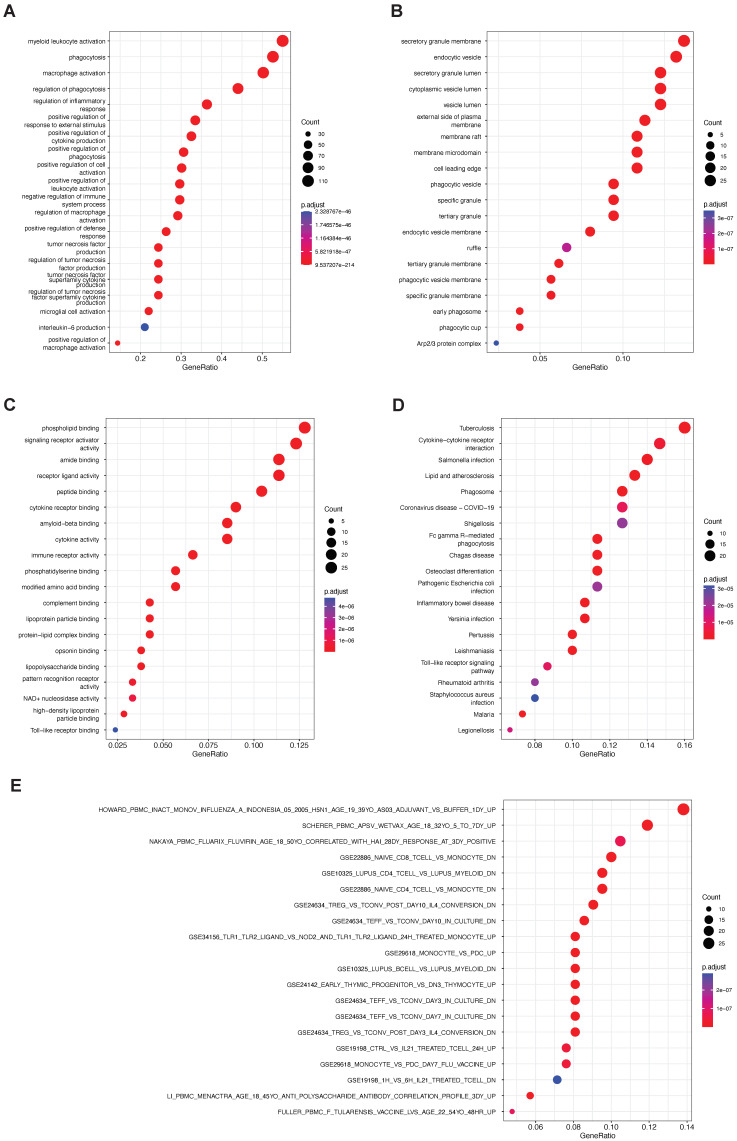
Functional enrichment analysis of phagocytic factors. (**A**) GOBP, gene ontology biological process; (**B**) GOCC, gene ontology cellular component; (**C**) GOMF, gene ontology molecular function; (**D**) KEGG, Kyoto Encyclopedia of Genes and Genomes; (**E**): set of immune-related genes.

Next, we focused on the functions of phagocytosis regulators. Using the R package clusterProfiler to carry out the enrichment analysis of GO and KEGG, we found that the phagocytosis factors were not only in GOBP (Figure 2A), GOCC (Figure 2B), GOMF (Figure 2C), or KEGG (Figure 2D). The pathway was not only significantly associated with cell membrane surface related components, but also involved in numerous immune-related gene sets. Enrichment analysis showed that phagocytosis factors were strongly correlated with T cells and monocytes (Figure 2E).

**Figure 3:**
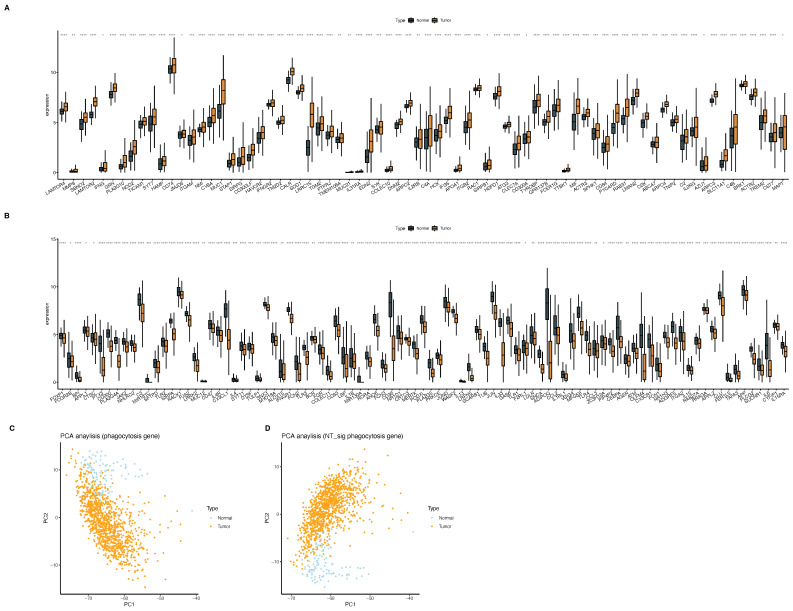
Differences in phagocytic factor expression between normal and tumor samples. (**A**) The expression level was higher in tumors than in normal tissues, (**B**) the expression level in normal tissues was higher than that in tumor samples, (**C**) PCA of all phagocytosis factors, and (**D**) PCA of normal and tumor differentially expressed phagocytic factors. Note, “e^−number^ represents 10^−number^” (* *p* < 0.05, ** *p* < 0.01,*** *p* < 0.001, **** *p* < 0.0001).

We focused on the expression of phagocytosis regulators in breast cancer versus standard samples. We used the Wilcoxon test to assess the differences in the expression of phagocytosis factors between tumor and normal samples. We found that 167 phagocytosis factors significantly differed in expression between tumor and normal samples (Supplementary Table S2). Among them, 74 genes were highly expressed (Figure 3A) and 93 genes were lowly expressed (Figure 3B) in tumors. Furthermore, we found that both the total phagocytosis factors and the differential phagocytosis factors between tumor and normal could distinguish normal samples from tumor samples (Figure 3C,D).

**Figure 4:**
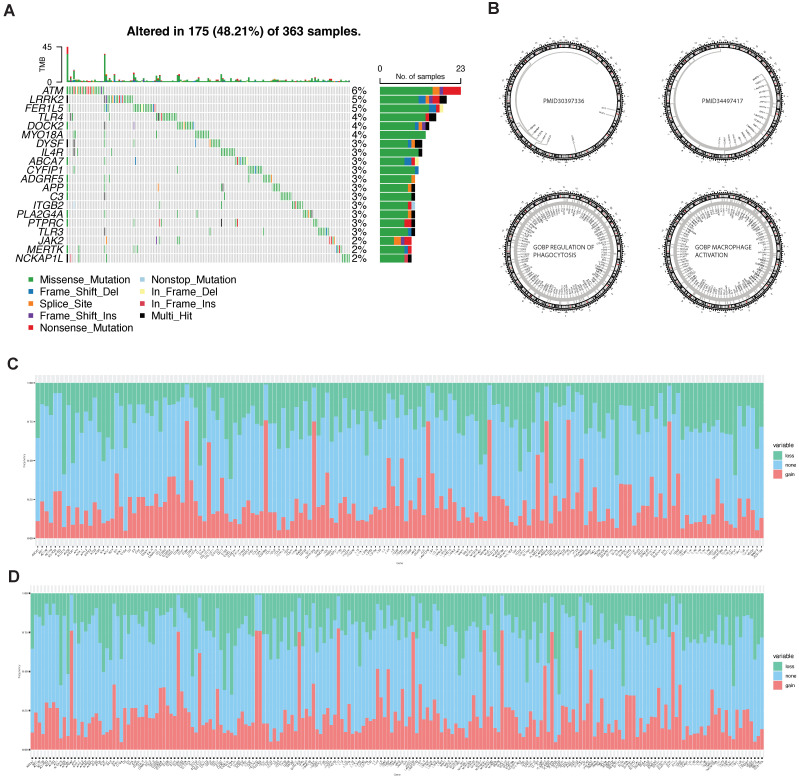
SNV and CNV of phagocytic factors. (**A**) Mutation rate top 20 phagocytic factors, (**B**) chromosome distribution of phagocytic factors according to different sources, marked in the center of circles, (**C**) the CNV of the mutant phagocytic factor, and (**D**) phagocytosis of CNV.

After that, we focused on genomic alterations in phagocytosis regulators. Based on TCGA mutation and CNV data, we obtained the overall mutation (Figure 4A) and CNV (Figure 4C,D) profiles of phagocytosis factors. Among all 235 phagocytosis factors, SNV occurred in 167 genes (Figure 4A, the top 20 genes with mutation rate). In terms of CNV, 206 genes showed CNV (Figure 4D). All mutated genes also developed CNV (Figure 4C).

**Figure 5:**
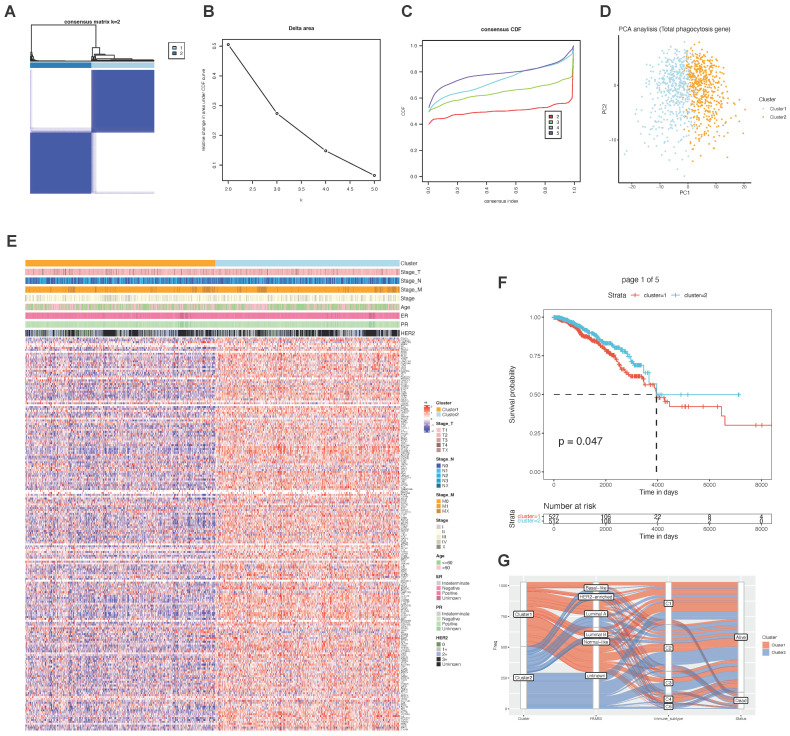
Sample clustering based on phagocytic factors. (**A**–**C**) Clustering results and parameters of unsupervised clustering, (**D**) PCA of phagocytosis factors, (**E**) expression of phagocytic factors and distribution of related clinical information among different clusters, (**F**) prognostic differences between the two groups, and (**G**) association of phagocytic subsets with related clinical features.

Next, we performed unsupervised clustering of TCGA-BRCA samples using the factors used for phagocytosis. We found that the samples could be divided into two categories (Figure 5A). There were significant differences between the different classes (Figure 5D). Phagocytosis factor expression was generally higher in cluster 2 than in cluster 1, while there were no significant differences in the clinical characteristics between the two classes (Table 2). However, there were significant prognostic differences between the two subgroups (Figure 5F). In normal-like samples, cluster 1 occupied the majority, and in the immune subtype of C1, cluster 1 occupied the majority (Figure 5G).

### 2.3. Identification and Characterization of Prognostic-Related Phagocytosis Regulators

**Figure 6:**
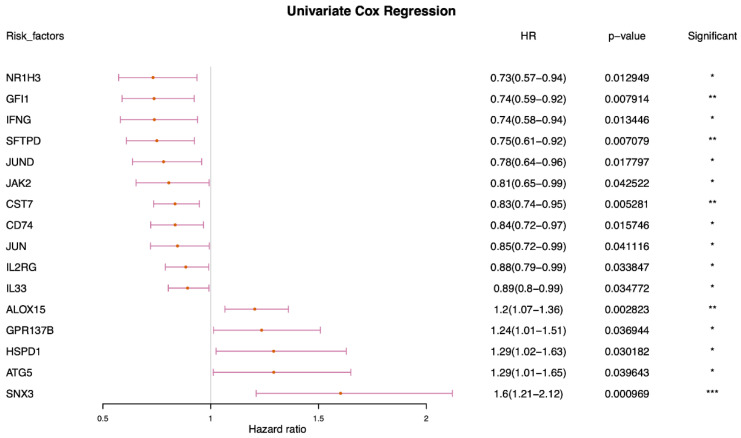
Univariate Cox analysis of phagocytosis regulatory factors. (* *p* < 0.05, ** *p* < 0.01, *** *p* < 0.001).

**Figure 7:**
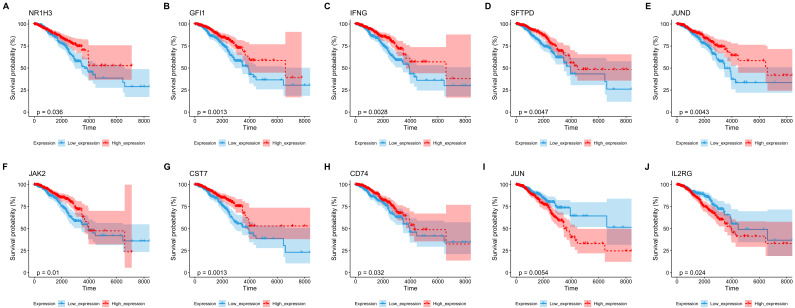
Of the 16 phagocytosis regulators, 10 genes had significant prognostic differences between the samples grouped by the median expression level of high and low expression. (**A**) *NR1H3*; (**B**) *GFI1*; (**C**) *IFNG*; (**D**) *SFTPD*; (**E**) *JUND*; (**F**) *JAK2*; (**G**) *CST7*; (**H**) *CD74*; (**I**) *JUN*; (**J**) *IL2RG*.

First, based on TCGA expression data and survival information, a univariate Cox screening of prognosis-related phagocytosis regulators was performed using the phagocytosis regulators. Sixteen regulators of phagocytosis were identified that were significantly associated with prognosis in univariate Cox analysis (Figure 6). Among the 16 regulators, 10 genes with significant prognostic values were selected to group the patients. The survival outcomes based on ten of these factors were presented (Figure 7).

### 2.4. Signature Construction of Phagocytosis Regulators

**Figure 8:**
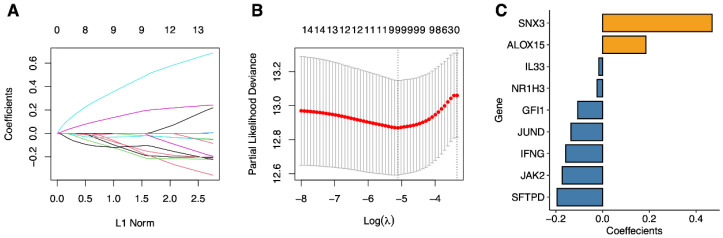
Lasso regression analysis. (**A**) The LASSO coefficient profile of OS-related genes, and an imaginary perpendicular line was drawn at the value chosen by 10-fold cross-validation. It reflects the association between coefficients and the L1 norm. (**B**) The tuning parameters (log λ) of OS-related proteins were selected to cross-verify the error curve. According to the minimal and 1-se criterion, imaginary perpendicular lines were drawn at the optimal value (**C**) Lasso coefficients for 9 genes.

Next, based on 16 prognostic-related genes, we used lasso Cox to remove redundant genes and constructed a phagocytosis-regulation-related signature (Figure 8). Figure 8C shows the screened genes and their coefficients. The details are displayed in Table 3.

### 2.5. The Phagocytosis Regulator Signature Is Related to the Prognosis and Clinical Characteristics of Patients

**Figure 9:**
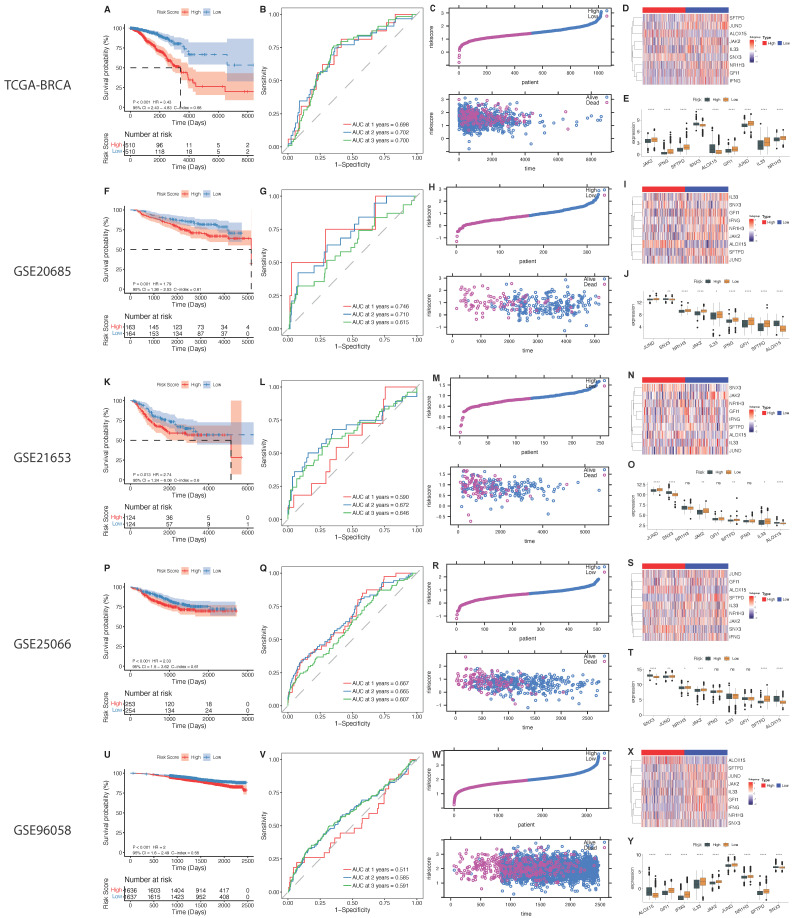
Identification of 9 genes with prognostic ability. (**A**–**E**) TCGA; (**F**–**J**), GSE20685; (**K**–**O**), GSE21653; (**P**–**T**), GSE25066; (**U**–**Y**), GSE96058; (**A**,**F**,**K**,**P**,**U**), OS analysis; (**B**,**G**,**L**,**Q**,**V**), 1, 2, and 3 year AUC; (**C**,**H**,**M**,**R**,**W**), sample risk core statistics (top), survival time and sample risk core statistics (bottom). (**D**,**I**,**N**,**S**,**X**) Expression heat map of 9 genes; (**E**,**J**,**O**,**T**,**Y**) expression difference between high and low-risk core groups of 9 genes. (* *p* < 0.05, ** *p* < 0.01, *** *p* < 0.001, **** *p* < 0.0001, and ns *p* value > 0.05).

For the nine significant prognostic genes reported by the lasso regression analysis outlined above, we performed energy efficiency analysis and prognostic power analysis on the training and validation sets. The samples were divided into high- and low-risk groups based on the median cutoff of the signature, and we found that in the TCGA dataset, these nine genes had significant prognostic power (Figure 9A), and the AUC at 1–3 years were all higher than 0.698 (Figure 9B). In the GSE20685, GSE21653, GSE25066, GSE96058 datasets, all nine of these genes had significant prognostic power (Figure 9F–V).

**Figure 10:**
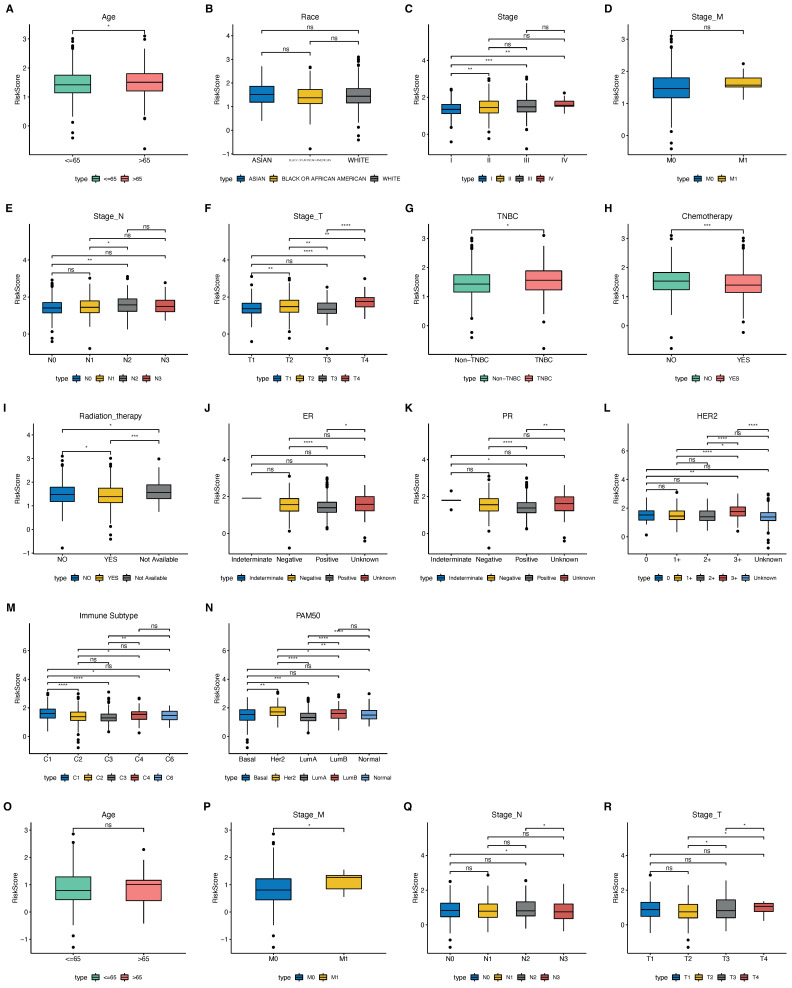
Clinical feature signature differences: (**A**–**N**) TCGA dataset; (**O**–**R**), G SE20685 dataset. (* *p* < 0.05, ** *p* < 0.01, *** *p* < 0.001, **** *p* < 0.0001, and ns *p* value > 0.05).

Based on the clinical features of the training set TCGA (Figure 10A–N) and the validation set GSE20685 (Figure 10O–R), the differences in the signatures of different clinical features are shown in Figure 10.

**Figure 11:**
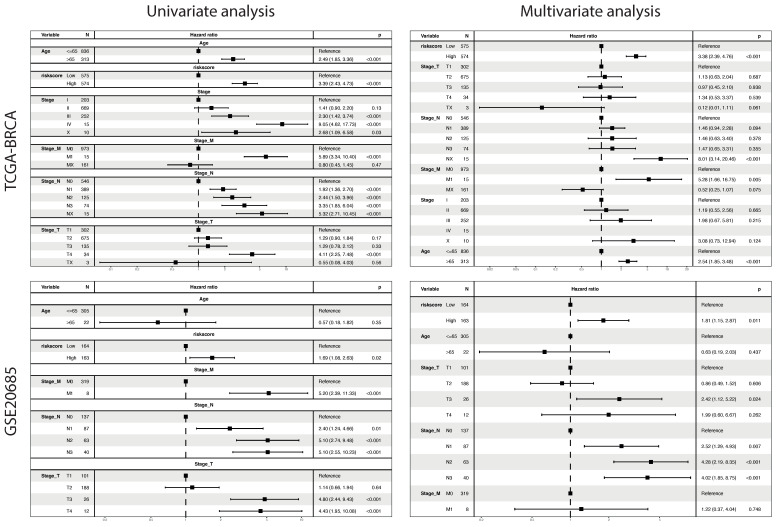
Signature is an independent prognostic factor of survival.

The signature was validated as an independent prognostic factor using univariate and multivariate Cox analyses (Figure 11).

**Figure 12:**
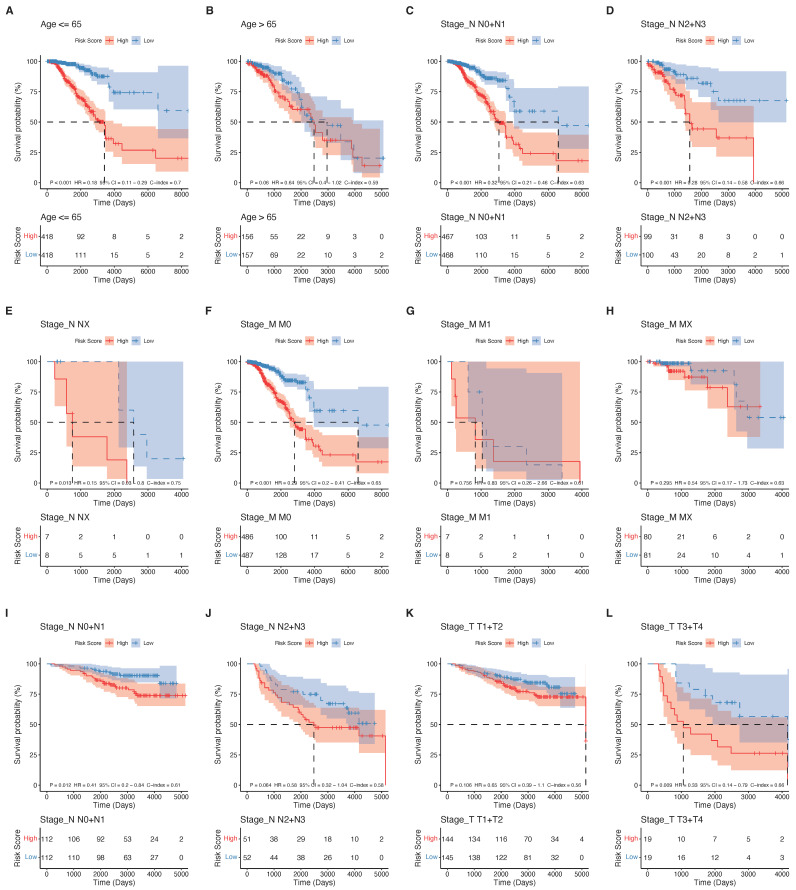
Predictive efficacy analysis of the signature.

For the clinical features that were significant in both univariate and multivariate Cox analyses in the above analysis, the clinical features were grouped in the TCGA dataset to explore the prognostic efficacy of the signature (Figure 12).

**Figure 13:**
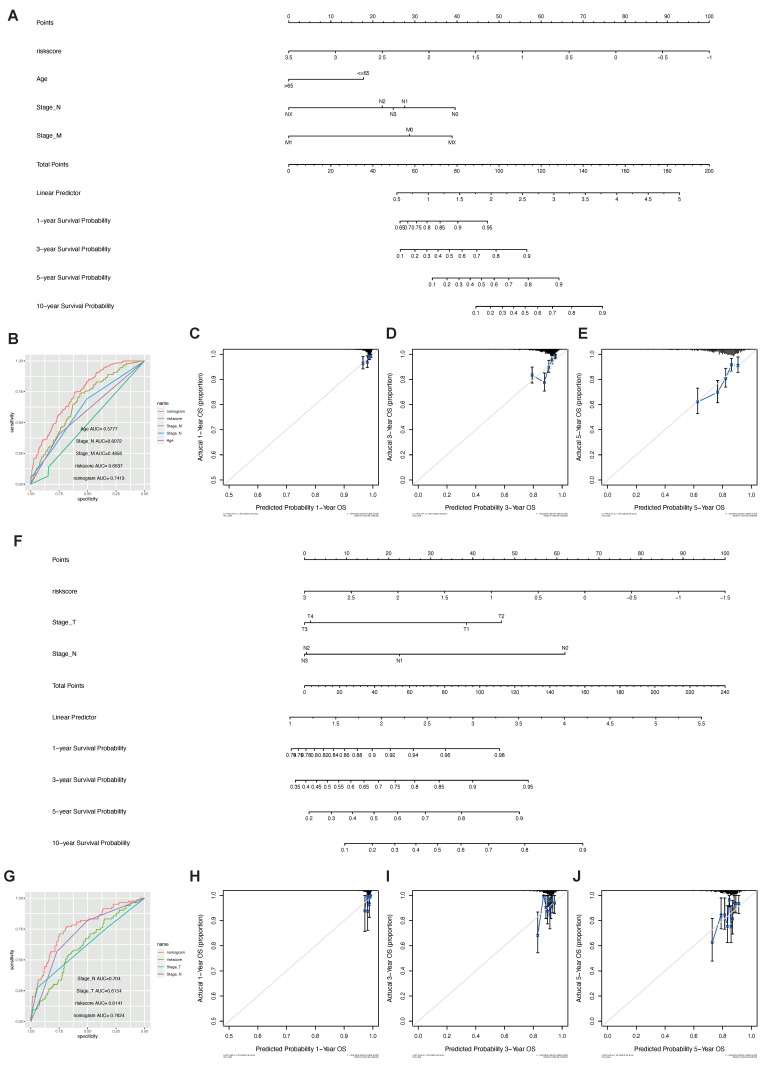
Nomogram and model performance. (**A**–**E**) TCGA dataset; (**F**–**J**) GSE20685 dataset. (**A**,**F**) Nomogram analysis of the predictive power of different clinical indicators and risk score; (**B**,**G**) ROC curves of different clinical features and risks core predicting OS. Model calibration curves for 1 year (**C**,**H**), 3 years (**D**,**I**), and 5 years (**E**,**J**).

The results showed that the nomogram had high precision in the calibration analysis. In the training (Figure 13A) and validation (Figure 13F) cohorts, the nomograms showed the strongest survival predictor compared with the other clinicopathological features (Figure 13B,G).

### 2.6. The Phagocytosis Regulator Signature Is Related to the Patient’s Immune Microenvironment and Immunotherapy

**Figure 14:**
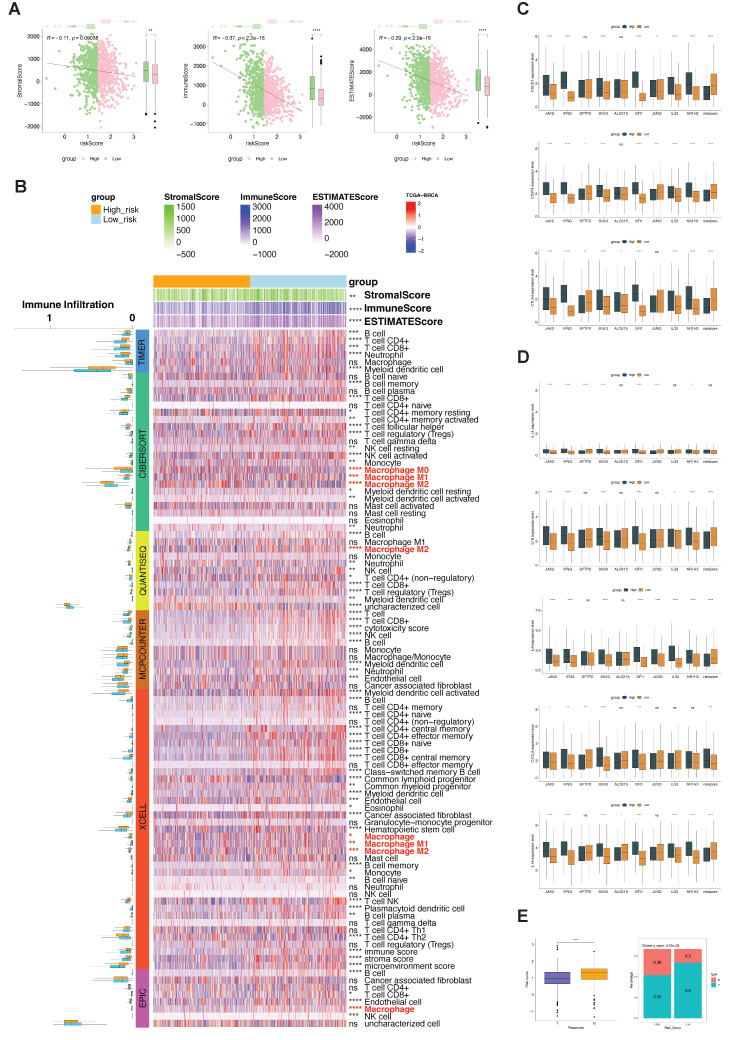
Immune correlates of phagocytosis factor signatures. (**A**) Correlation between risk score, matrix score, immune score, and estimate score; (**B**) differences in immune cell infiltration between risk score groups; (**C**) immune checkpoints; (**D**) correlated expression of proinflammatory factors, phagocytosis factors, and risk score; (**E**) correlation of phagocytosis factor risk score with drug treatment response in the immunotherapy cohort. (* *p* < 0.05, ** *p* < 0.01, *** *p* < 0.001, **** *p* < 0.0001, and ns *p* value > 0.05).

We first focused on the association of phagocytosis regulator signatures with immune scores and immune infiltrating cells. Using the estimate package, we calculated the immune, matrix, and estimate scores for TCGA-BRCA samples. For the matrix, immune, or estimate score, there was a significant negative correlation with the risk score of the phagocytosis regulator signature (Figure 14A). We used six methods, i.e., timer, cibersort, quantiseq, MCP counter, XCell, and EPIC analysis, to assess immune cell infiltration in TCGA-BRCA samples. We found significant infiltration differences with regard to macrophages between samples grouped with high and low phagocytosis regulator signatures in all four methods, and the trends in immune infiltration analyzed by all methods were consistent with the trend in immune scores reported by the estimate algorithm (Figure 14B).

Subsequently, using the immune checkpoint genes, PDCD1, PD-L1 (CD274), and C TLA-4 and proinflammatory factors, interleukin-1 alpha (IL-1 alpha), IL-1 β, IL-6, IL-8/CXCL8, and IL-18, we explored the high and low expression of genes included in the signature based on the high and low groups of signatures. We also analyzed the differences in the expression of immune checkpoints (Figure 14C) and the expression of pro-inflammatory factors (Figure 14C,D). We found that there were significant differences in the expression of the above genes between the high and low signature groups and the groups with high versus low gene expression.

Furthermore, in clear cell renal cell carcinoma (PMID32472114) [9], we analyzed the differences between the high and low signature groups and the drug response before and after treatment. We found that there were significant differences in the risk scores among different groups in response to the drug, and the risk scores in the nonresponse group were significantly higher than that in the responsive group (Figure 14E left). In the high- and low-risk score groups, the number of patients with different drug responses was also significantly different (Figure 14E right, chi-square test, *p* < 0.0001).

### 2.7. Genomic Alteration of the Phagocytosis Regulator Signature and Drug Response

Based on the mutation data of TCGA-BRCA, we analyzed the mutation differences in the high- and low-risk score groups. We observed that the mutation rate of the TP53 gene was significantly higher in the high-risk group than in the low-risk group (Figure S3A). After analyzing CNV data, we found that the incidence of CNV was significantly higher in the high-risk group than in the low-risk group (Figure S3B,C). In the statistical analysis using TMB, there was no significant prognostic difference between the high- and low-risk groups divided by the median TMB (Figure S3D). Still, there was a significant difference in the risk score between the two groups. The risk score of the high TMB group was significantly higher than that of the group with a low TMB (Figure S3E).

Next, we explored the relationship between risk score and drug tolerance using GDSC and CCLE data. We performed Spearman correlation analysis on risk score and IC50 values in GDSC and CCLE data, respectively. By counting the Spearman correlations between risk score and IC50 values in both databases >0.2 or <−0.2, and the *p* for drugs < 0.05, we finally identified 27 drugs associated with THE risk score. Notably, none of the drugs showed a negative correlation between IC50 and risk score, suggesting that risk scores have consistent universality in predicting chemotherapeutic drug resistance (Figure S3F).

## 3. Discussion

The classical adaptive response of macrophages includes tolerance, initiation, and a wide range of activation states, including M1 or M2 [10,11]. Macrophages produce key immunosuppressive mediators, including cytokines (IL-10), enzymes involved in amino acid metabolism (arginase), prostaglandins, and triggers of immune checkpoint blockade in T and NK cells (e.g., PD-L1,VISTA) [12,13,14]. Macrophage phagocytic factor is an essential component of the complex gene network that controls macrophage polarization, activation, and plasticity [15,16,17]. The combination of macrophage phagocytic factor and novel immunotherapeutic regimens shows great promise for treating breast cancer [18,19]. Therefore, it may be advantageous to identify macrophage phagocytic-factor-related biomarkers that help distinguish breast cancer patients premised on the benefits they derive from immunotherapy. Here, we demonstrated that the expression of macrophage phagocytic factors is closely associated with the prognosis and tumor microenvironment of breast cancer. We classified two macrophage phagocytic factor subgroups by unsupervised cluster analysis, and found that the high expression of macrophage phagocytic factor was associated with favorable clinical outcomes and a high level of immune cell infiltration. Moreover, we constructed and validated a prognostic risk signature with nine selected macrophage phagocytic factor genes, stratifying breast cancer patients into high- and low-risk cohorts. In addition, this risk signature showed a high predictive value in terms of OS and drug resistance to chemotherapy as an independent prognostic indicator for breast cancer.

The macrophage phagocytic factor gene involved in this study was identified through an extensive literature survey, and a total of 214 related genes were extracted. In our analysis, 28 genes were related to macrophage enrichment, and the other 167 phagocytic factors showed significant expression differences between tumor and normal samples.

TME includes immune system components such as macrophages and lymphocytes, blood-vessel-forming cells, fibroblasts, myofibroblasts, mesenchymal stem cells, adipocytes, and the extracellular matrix (ECM) [20]. Among these cells, tumor-associated macrophages (TAM) are the main components of TME in breast cancer [21]. Macrophages show a high degree of plasticity in response to various external signals and are involved in congenital and adaptive immune responses to control numerous factors of the TME [22]. Clinicopathological studies have shown that TAM accumulation in tumors is associated with poor clinical outcomes. In human breast cancer, a high TAM density is associated with poor prognosis [23,24]. Over the years, studies on the role of TAM in the progression of breast cancer have determined that TAM can induce angiogenesis, reshape the tumor extracellular matrix to aid invasion, mimic breast cancer cells to evade the host immune system, and recruit immunosuppressive white blood cells into the tumor microenvironment [25,26]. These observations make TAM an attractive target for therapeutic interventions by targeting various aspects of its function. In line with this evidence, our study identified two macrophage phagocytic factor subgroups by consensus clustering, and the MPPF-high subgroup was associated with the immune-hot phenotype. In contrast, the MPPF-low subgroup was referred to as the immune-cold phenotype.

## 4. Methods and Materials

### 4.1. Data Download

This project involved two parts: a training set and a validation set. The training set included the TCGA-BRCA dataset, and the validation set contained data from four GEO datasets: GSE20685, GSE21653, GSE25066, and GSE96058. In addition, the immunotherapy dataset EGAS00001004353 was prepared for later analysis.

We used the TCGA-BRCA dataset to collect transcriptomic, mutational, copy number variation, and informative clinical data. The data source was Xena [27], and the download address was https://portal.gdc.cancer.gov/ (accessed on 16 March 2022). Log2 (TPM + 1) was used for all analyses involving TCGA transcript data. GEO data were downloaded from https://www.ncbi.nlm.nih.gov/geo/ (accessed on 16 March 2022) [28]. Dataset EGAS00001004353 was downloaded from sub.ega-archive.org/datasets/ (accessed on 16 March 2022) [29].

### 4.2. Data Overview

The data are summarized in Table 4.

### 4.3. Pathway Enrichment Analysis

Pathway enrichment analysis was performed using the R package clusterProfiler [30], and ssGSEA enrichment was performed using the GSVA package [31] with default parameters.

### 4.4. Analysis of CNV and SNV

SNV analysis was performed based on the R package maftools package [32]. The mutation situation of the TCGA-BRCA dataset was analyzed using the default parameters, and the statistics of the mutation results were directly generated by the oncoplot function of the maftools package.

CNV was performed using Gistic2 [33]. The specific parameters used were set as follows: ta:0.1, armpeel:1, brlen:0.7, cap:1.5, conf:0.75, td:0.1, enegistic:1, gcm:extreme, js:4, maxseg:2000, qvt:0.25, rx:0, savegene:1.

### 4.5. Immune Infiltration Analysis

Samples were subjected to immune infiltration analysis using the R packages C IBERSORT [34], Quantiseq [35], Xcell [36], EPIC and MCPCounter [37]. All analysed parameters are default parameters. The timer was performed using the online tool Timer 2.0.

### 4.6. Calculation of Tumor Purity, Stromalscore, Immunescore

Tumor purity, stromal score, and immune score were calculated using the R package estimate [38]. Here, we ran it with the parameter platform = “illumine”.

### 4.7. Consistent Clustering

Hub-based mRNA consensus clustering of tumor samples in the TCGA-BRCA dataset was performed using the consensusClusterPlus package [39]. For the TCGA-BRCA data, we used the R package GeoTcgaData to perform TPM conversion on the count data. Consistent clustering was performed using the pam method, Canberra distance and seed = 1, and k. max = 5 parameters.

### 4.8. Construction of Immune Infiltrating Cell Marker Scoring System Is Capable of Evaluating Prognosis

We used the cv.glmnet function of the glmnet package [40] to perform lasso analysis on the samples and the corresponding genes. The parameter used in Lasso analysis was family = “cox”, and the level of significance was based on *p* < 0.05. Genes with coefficients not equal to 0 were screened and included in the final model. We defined the risk score as the sum of the product of the lasso coefficient and the gene expression level.

### 4.9. KM Survival Analysis

KM survival analysis and plotting were performed using the R packages survival and ggsurvplot (https://CRAN.R-project.org/package=survminer (accessed on 16 March 2022)). Analysis was performed using default parameters. AUC was analyzed using the R package timeROC (https://CRAN.R-project.org/package=timeROC (accessed on 16 March 2022)).

### 4.10. Correlation Analysis between Risk Score and Drug Sensitivity

We downloaded the drug sensitivity data for approximately 1000 cancer cell lines from Genomics of Cancer Drug Sensitivity (GDSC) (cancerrxgene.org). Taking the antitumor drug IC50 in cancer cell lines as the drug response index, we used Spearman correlation analysis to calculate the correlation between drug sensitivity and the risk score and considered the absolute value of Rs > 0.2 and *p*-value < 0.05 as significantly related. The same approach was also performed on the CCLE data (https://sites.broadinstitute.org/ccle/datasets (accessed on 16 March 2022)).

### 4.11. Principal Component Analysis (PCA) Analysis

PCA analysis was performed using the R built-in function prcomp.

### 4.12. Drawing

Circus diagrams were drawn using the R package circlize (https://CRAN.R-project.org/package=circlize (accessed on 16 March 2022)). Sankey diagrams were drawn using the R package ggalluvial. Nomograms were made using the R package nomogram [41].

### 4.13. Model Calibration Curve

Calibration curves were analyzed using the R package rms [42].

### 4.14. Statistical Tests

The Wilcoxon test was used for comparison between groups. The validity of the model was verified with the receiver operating characteristic (ROC) curve. We divided the samples by the median risk score. Survival curves were drawn using the Kaplan–Meier method for predictive analysis, and the log-rank test was used to determine the significance of differences. To assess whether the risk score was an independent predictor, we performed a multivariate cox regression model analysis with age, sex, and stage as variables. Correlation analysis was performed using Spearman or Pearson correlation. All statistical analyses were two-sided, and *p* < 0.05 was considered statistically significant (* *p* < 0.05, ** *p* < 0.01, *** *p* < 0.001, **** *p* < 0.0001, and ns *p* value > 0.05).

## 5. Conclusions

In conclusion, in our study, we constructed a meaningful signature based on the macrophage phagocytic factor genes for breast cancer patients, which showed significant value in predicting the OS of breast cancer patients. These results indicate the need for future research on and immune-therapy-based interventions for breast cancer patients.

## Figures and Tables

**Table 1:** Macrophage enrichment score was correlated with phagocyte factor.

Gene	Rho	*p*-Value
*FCER1G*	0.74895132	7.40 × 10^−197^
*ITGB2*	0.73940791	2.53 × 10^−189^
*CD300LF*	0.73874914	8.16 × 10^−189^
*NCKAP1L*	0.71075865	1.44 × 10^−168^
*HAVCR2*	0.7035216	1.01 × 10^−163^
*CD74*	0.69829577	2.59 × 10^−160^
*AIF1*	0.69692323	1.98 × 10^−159^
*C1QA*	0.68336001	5.75 × 10^−151^
*IL2RG*	0.68100437	1.52 × 10^−149^
*DOCK2*	0.66931193	1.13 × 10^−142^
*TYROBP*	0.66309895	3.77 × 10^−139^
*CST7*	0.645747	9.63 × 10^−130^
*FGR*	0.64426929	5.71 × 10^−129^
*IL15RA*	0.64193309	9.33 × 10^−128^
*SIRPG*	0.62837445	6.41 × 10^−121^
*GFI1*	0.61166452	6.03 × 10^−113^
*CTSC*	0.60386822	2.19 × 10^−109^
*PTPRC*	0.60132084	3.03 × 10^−108^
*HCK*	0.59651545	4.05 × 10^−106^
*CD300A*	0.58598236	1.39 × 10^−101^
*NMI*	0.58263075	3.57 × 10^−100^
*IL10*	0.56451739	7.77 × 10^−93^
*FCN1*	0.55325685	1.69 × 10^−88^
*FCGR2B*	0.54762828	2.16 × 10^−86^
*SIRPB1*	0.52573722	1.44 × 10^−78^
*C5AR1*	0.52253377	1.80 × 10^−77^
*C3*	0.51378244	1.57 × 10^−74^
*IFNG*	0.5026317	6.58 × 10^−71^

**Table 2:** Statistical test of clinical characteristics among subgroups.

Clinical Traits	*p*-Value
Stage_T	0.91681495
Stage_N	0.75402253
Stage_M	0.82725935
Stage	0.60040185
Age	1
ER	0.77282999
PR	0.88454944
HER2	0.91681495

**Table 3:** Lasso coefficient.

Symbol	Coefficient
*JAK2*	−0.1728154
*IFNG*	−0.1576737
*SFTPD*	−0.1945068
*SNX3*	0.46774561
*ALOX15*	0.18521293
*GFI1*	−0.1071469
*JUND*	−0.1352052
*IL33*	−0.0164065
*NR1H3*	−0.0237538

**Table 4:** The details of database and survival outcomes in this study.

GEO Database	Platform	Platform Access Number	Total Number	Overall Survival
TCGA-BRCA	RNA-Seq	-	1211	Alive:891
				Dead:135
GSE20685	Affymetrix Human Genome U133 Plus 2.0 Array	GPL570	327	Alive:244
				Dead:83
GSE21653	Affymetrix Human Genome U133 Plus 2.0 Array	GPL570	266	Alive:169
				Dead:83
GSE25066	Affymetrix Human Genome U133A Array	GPL96	508	Alive:397
				Dead:111
GSE96058	Illumina HiSeq 2000	-	3409	Alive:2937
				Dead:336
EGAS00001004353	RNA-Seq	-	726	Alive:422
				Dead:463

## Data Availability

All data generated or analyzed during this study are included in this published article and its Supplementary Materials.

## Supplementary Materials

The following supporting information can be downloaded at: https://www.mdpi.com/article/10.3390/ijms231810312/s1.

## References

[1] Yersal O., Barutca S. (2014). Biological subtypes of breast cancer: Prognostic and therapeutic implications. World J. Clin. Oncol..

[2] Goldhirsch A., Wood W.C., Gelber R.D., Coates A.S., Thürlimann B., Senn H.J. (2007). Progress and promise: Highlights of the international expert consensus on the primary therapy of early breast cancer 2007. Ann. Oncol..

[3] Alexander W. (2016). The Checkpoint Immunotherapy Revolution: What Started as a Trickle Has Become a Flood, Despite Some Daunting Adverse Effects; New Drugs, Indications, and Combinations Continue to Emerge. Pharm. Ther..

[4] Zhang Y., Zhang Z. (2020). The history and advances in cancer immunotherapy: Understanding the characteristics of tumor-infiltrating immune cells and their therapeutic implications. Cell. Mol. Immunol..

[5] Bai R., Li W., Du N., Cui J. (2019). Challenges of evaluating immunotherapy efficacy in solid tumors. Chin. J. Cancer Res..

[6] Kamber R.A., Nishiga Y., Morton B., Banuelos A.M., Barkal A.A., Vences-Catalán F., Gu M., Fernandez D., Seoane J.A., Yao D. (2021). Inter-cellular CRISPR screens reveal regulators of cancer cell phagocytosis. Nature.

[7] Haney M.S., Bohlen C.J., Morgens D.W., Ousey J.A., Barkal A.A., Tsui C.K., Ego B.K., Levin R., Kamber R.A., Collins H. (2018). Identification of phagocytosis regulators using magnetic genome-wide CRISPR screens. Nat. Genet..

[8] Charoentong P., Finotello F., Angelova M., Mayer C., Efremova M., Rieder D., Hackl H., Trajanoski Z. (2017). Pan-cancer Immunogenomic Analyses Reveal Genotype-Immunophenotype Relationships and Predictors of Response to Checkpoint Blockade. Cell Rep..

[9] Braun D.A., Hou Y., Bakouny Z., Ficial M., Sant’ Angelo M., Forman J., Ross-Macdonald P., Berger A.C., Jegede O.A., Elagina L. (2020). Interplay of somatic alterations and immune infiltration modulates response to PD-1 blockade in advanced clear cell renal cell carcinoma. Nat. Med..

[10] Raggi F., Pelassa S., Pierobon D., Penco F., Gattorno M., Novelli F., Eva A., Varesio L., Giovarelli M., Bosco M.C. (2017). Regulation of Human Macrophage M1–M2 Polarization Balance by Hypoxia and the Triggering Receptor Expressed on Myeloid Cells-1. Front. Immunol..

[11] Locati M., Curtale G., Mantovani A. (2020). Diversity, Mechanisms, and Significance of Macrophage Plasticity. Annu. Rev. Pathol..

[12] Groth C., Hu X., Weber R., Fleming V., Altevogt P., Utikal J., Umansky V. (2019). Immunosuppression mediated by myeloid-derived suppressor cells (MDSCs) during tumour progression. Br. J. Cancer.

[13] Grzywa T.M., Sosnowska A., Matryba P., Rydzynska Z., Jasinski M., Nowis D., Golab J. (2020). Myeloid Cell-Derived Arginase in Cancer Immune Response. Front. Immunol..

[14] Ceci C., Atzori M.G., Lacal P.M., Graziani G. (2020). Targeting Tumor-Associated Macrophages to Increase the Efficacy of Immune Checkpoint Inhibitors: A Glimpse into Novel Therapeutic Approaches for Metastatic Melanoma. Cancers.

[15] Shapouri-Moghaddam A., Mohammadian S., Vazini H., Taghadosi M., Esmaeili S.-A., Mardani F., Seifi B., Mohammadi A., Afshari J.T., Sahebkar A. (2018). Macrophage plasticity, polarization, and function in health and disease. J. Cell. Physiol..

[16] Orecchioni M., Ghosheh Y., Pramod A.B., Ley K. (2019). Macrophage Polarization: Different Gene Signatures in M1(LPS+) vs. Classically and M2(LPS-) vs. Alternatively Activated Macrophages. Front. Immunol..

[17] Palma A., Jarrah A.S., Tieri P., Cesareni G., Castiglione F. (2018). Gene Regulatory Network Modeling of Macrophage Differentiation Corroborates the Continuum Hypothesis of Polarization States. Front. Physiol..

[18] Kumari N., Choi S.H. (2022). Tumor-associated macrophages in cancer: Recent advancements in cancer nanoimmunotherapies. J. Exp. Clin. Cancer Res..

[19] Reis-Sobreiro M., Teixeira da Mota A., Jardim C., Serre K. (2021). Bringing Macrophages to the Frontline against Cancer: Current Immunotherapies Targeting Macrophages. Cells.

[20] Wang M., Zhao J., Zhang L., Wei F., Lian Y., Wu Y., Gong Z., Zhang S., Zhou J., Cao K. (2017). Role of tumor microenvironment in tumorigenesis. J. Cancer.

[21] Larionova I., Tuguzbaeva G., Ponomaryova A., Stakheyeva M., Cherdyntseva N., Pavlov V., Choinzonov E., Kzhyshkowska J. (2020). Tumor-Associated Macrophages in Human Breast, Colorectal, Lung, Ovarian and Prostate Cancers. Front. Oncol..

[22] Li X., Liu R., Su X., Pan Y., Han X., Shao C., Shi Y. (2019). Harnessing tumor-associated macrophages as aids for cancer immunotherapy. Mol. Cancer.

[23] Qiu S.-Q., Waaijer S.J.H., Zwager M.C., de Vries E.G.E., van der Vegt B., Schröder C.P. (2018). Tumor-associated macrophages in breast cancer: Innocent bystander or important player?. Cancer Treat. Rev..

[24] Zhao X., Qu J., Sun Y., Wang J., Liu X., Wang F., Zhang H., Wang W., Ma X., Gao X. (2017). Prognostic significance of tumor-associated macrophages in breast cancer: A meta-analysis of the literature. Oncotarget.

[25] Gil Del Alcazar C.R., Alečković M., Polyak K. (2020). Immune Escape during Breast Tumor Progression. Cancer Immunol. Res..

[26] Baghban R., Roshangar L., Jahanban-Esfahlan R., Seidi K., Ebrahimi-Kalan A., Jaymand M., Kolahian S., Javaheri T., Zare P. (2020). Tumor microenvironment complexity and therapeutic implications at a glance. Cell Commun. Signal..

[27] Goldman M.J., Craft B., Hastie M., Repečka K., McDade F., Kamath A., Banerjee A., Luo Y., Rogers D., Brooks A.N. (2020). Visualizing and interpreting cancer genomics data via the Xena platform. Nat. Biotechnol..

[28] Barrett T., Wilhite S.E., Ledoux P., Evangelista C., Kim I.F., Tomashevsky M., Marshall K.A., Phillippy K.H., Sherman P.M., Holko M. (2012). NCBI GEO: Archive for functional genomics data sets—Update. Nucleic Acids Res..

[29] Freeberg M.A., Fromont L.A., D’Altri T., Romero A.F., Ciges J.I., Jene A., Kerry G., Moldes M., Ariosa R., Bahena S. (2021). The European Genome-phenome Archive in 2021. Nucleic Acids Res..

[30] Wu T., Hu E., Xu S., Chen M., Guo P., Dai Z., Feng T., Zhou L., Tang W., Zhan L. (2021). clusterProfiler 4.0: A universal enrichment tool for interpreting omics data. Innovation.

[31] Hänzelmann S., Castelo R., Guinney J. (2013). GSVA: Gene set variation analysis for microarray and RNA-Seq data. BMC Bioinform..

[32] Mayakonda A., Lin D.C., Assenov Y., Plass C., Koeffler H.P. (2018). Maftools: Efficient and comprehensive analysis of somatic variants in cancer. Genome Res..

[33] Mermel C.H., Schumacher S.E., Hill B., Meyerson M.L., Beroukhim R., Getz G. (2011). GISTIC2.0 facilitates sensitive and confident localization of the targets of focal somatic copy-number alteration in human cancers. Genome Biol..

[34] Chen B., Khodadoust M.S., Liu C.L., Newman A.M., Alizadeh A.A. (2018). Profiling Tumor Infiltrating Immune Cells with CIBERSORT. Methods Mol. Biol..

[35] Finotello F., Mayer C., Plattner C., Laschober G., Rieder D., Hackl H., Krogsdam A., Posch W., Wilflingseder D., Sopper S. (2017). quanTIseq: Quantifying immune contexture of human tumors. BioRxiv.

[36] Aran D., Hu Z., Butte A.J. (2017). xCell: Digitally portraying the tissue cellular heterogeneity landscape. Genome Biol..

[37] Becht E., Giraldo N.A., Lacroix L., Buttard B., Elarouci N., Petitprez F., Selves J., Laurent-Puig P., Sautès-Fridman C., Fridman W.H. (2016). Estimating the population abundance of tissue-infiltrating immune and stromal cell populations using gene expression. Genome Biol..

[38] Yoshihara K., Shahmoradgoli M., Martínez E., Vegesna R., Kim H., Torres-Garcia W., Treviño V., Shen H., Laird P.W., Levine D.A. (2013). Inferring tumour purity and stromal and immune cell admixture from expression data. Nat. Commun..

[39] Wilkerson M.D., Hayes D.N. (2010). ConsensusClusterPlus: A class discovery tool with confidence assessments and item tracking. Bioinformatics.

[40] Simon N., Friedman J., Hastie T., Tibshirani R. (2011). Regularization Paths for Cox’s Proportional Hazards Model via Coordinate Descent. J. Stat. Softw..

[41] Zhang Z., Kattan M.W. (2017). Drawing Nomograms with R: Applications to categorical outcome and survival data. Ann. Transl. Med..

[42] Harrell F.E., Harrell M.F.E., Hmisc D.J.V.U. (2017). Package ‘rms’. https://github.com/harrelfe/rms.

