# Supplementary material for: Phagocytosis-Regulators-Based Signature to Predict the Prognosis and Chemotherapy Resistance for Breast Cancer Patients
Source: Int J Mol Sci. 2022 Sep 7;23(18):10312. doi: 10.3390/ijms231810312 (PMC9499262; doi:10.3390/ijms231810312)
Supplement: Supplementary file 1 [file ijms-23-10312-s001.zip › Supplement Figures S1-S3.pdf]

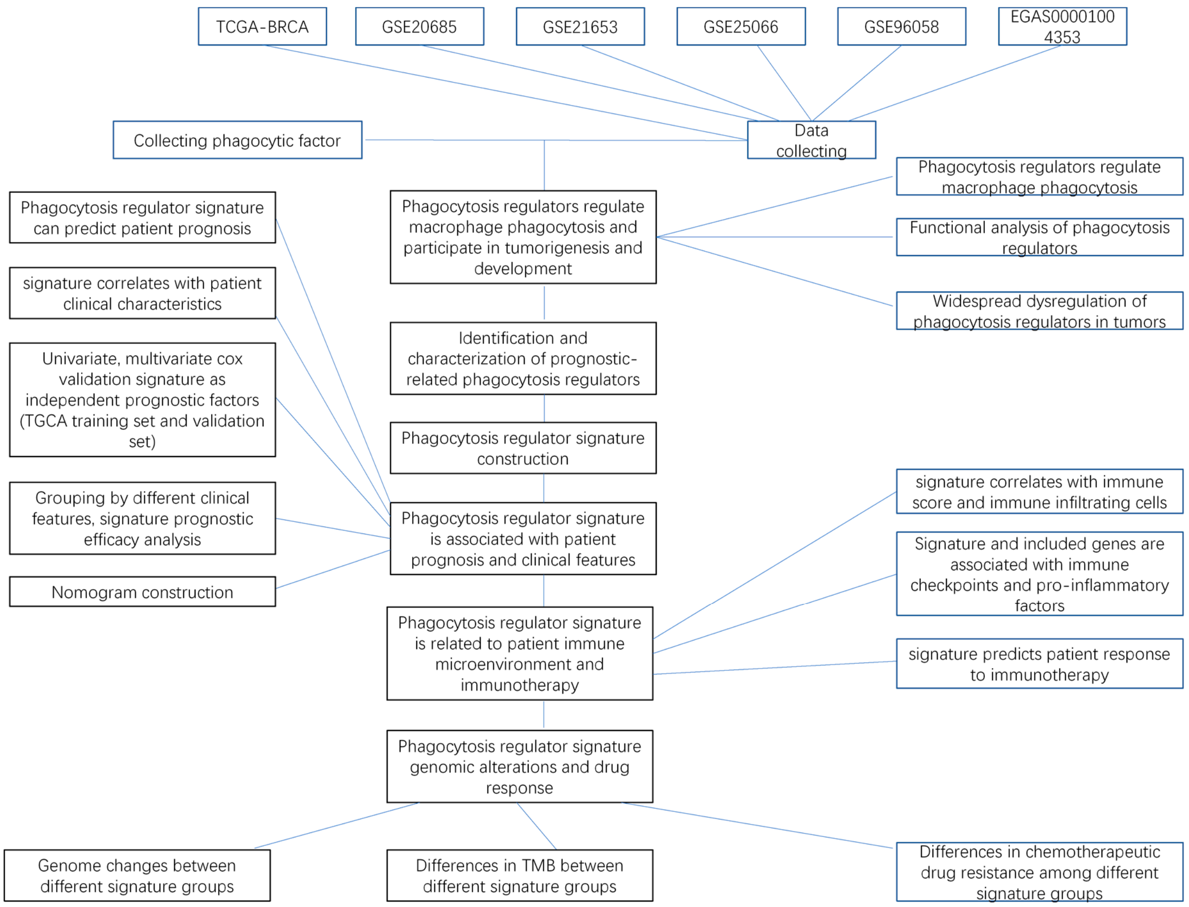

Figure S1. The flow chart of the overall study design.

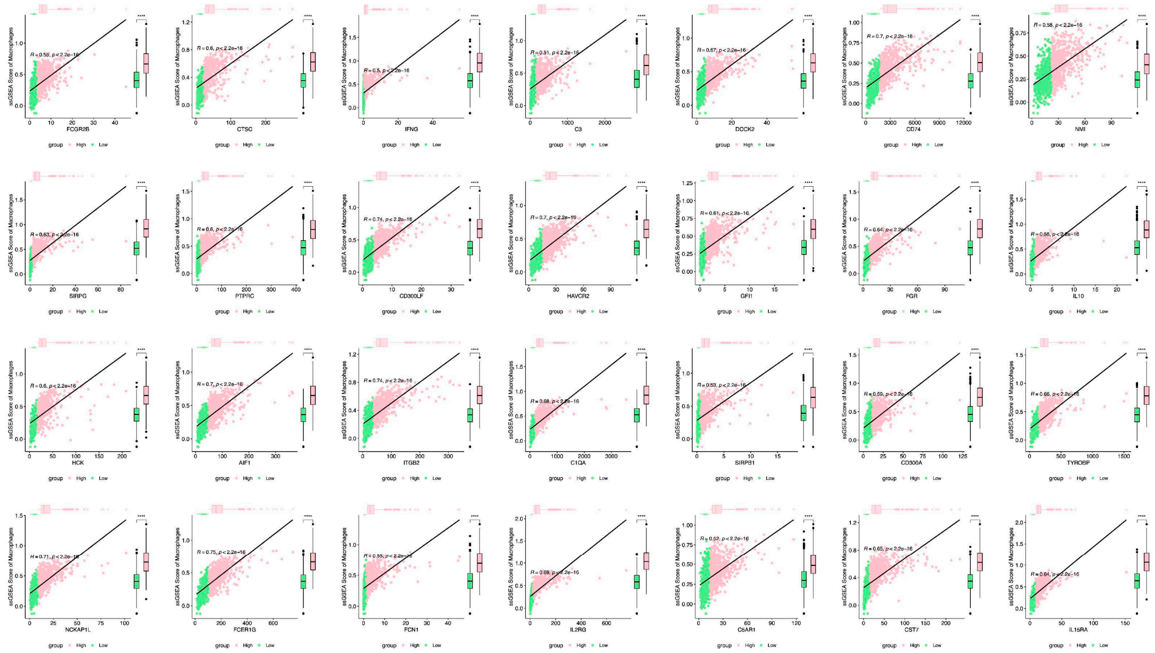

Figure S2. The expression of macrophage and macrophage enrichment score correlation analysis with other phagocytic factors.

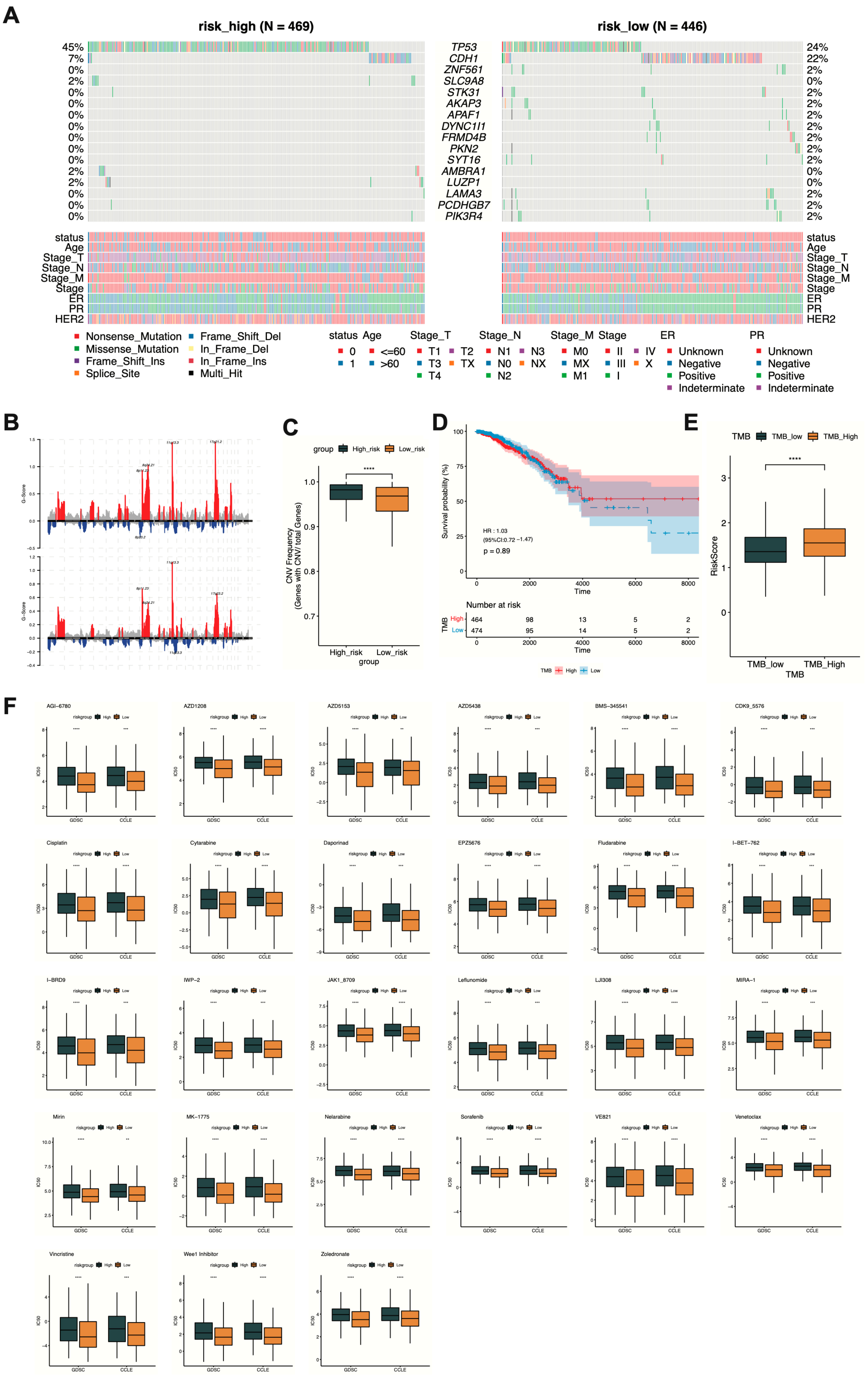
A
risk_high (N = 469)
risk_low (N = 446)
TP53
CDH1
ZNF561
SLC9A8
STK31
AKAP3
APAF1
DYNC1I1
FRMD4B
PKN2
SYT16
AMBRA1
LUZP1
LAMA3
PCDHGB7
PIK3R4
status
Age
Stage_T
Stage_N
Stage_M
Stage
ER
PR
HER2
Nonsense_Mutation
Missense_Mutation
Frame_Shift_Ins
Splice_Site
Frame_Shift_Del
In_Frame_Del
In_Frame_Ins
Multi_Hit
Unknown
Negative
Positive
Indeterminate
B
G-Score
C
group High_risk Low_risk
CNV Frequency (Genes with CNV/ total Genes)
High_risk
Low_risk
group
D
Survival probability (%)
HR : 1.03
(95%CI:0.72 - 1.47)
p = 0.89
Time
Number at risk
TMB
High
Low
E
TMB TMB_low TMB_High
RiskScore
TMB_low
TMB_High
TMB
F
AGI-6780
AZD1208
AZD5153
AZD5438
BMS-345541
CDK9_5576
Cisplatin
Cytarabine
Daporinad
EPZ5676
Fludarabine
I-BET-762
I-BRD9
IWP-2
JAK1_8709
Leflunomide
LJI308
MIRA-1
Mirin
MK-1775
Nelarabine
Sorafenib
VE821
Venetoclax
Vincristine
Wee1 Inhibitor
Zoledronate
riskgroup High Low
IC50
GDSC
CCLE

Figure S3. Genomics prediction of drug tolerance by phagocytosis factor risk score. A, SNV analysis between high and low risk groups; B, copy number variation statistics of high-risk group (top) and low-risk group (bottom); C, Differences in the incidence of copy number variation between high and low risk groups; D, Prognosis between high and low TMB groups; E, Differences in riskscore between high and low TNB groups; F, GDSC and CCEL for 27 drugs to determine which drug tolerance was significantly associated with riskscore.
