# Supplementary material for: Phagocytosis-Regulators-Based Signature to Predict the Prognosis and Chemotherapy Resistance for Breast Cancer Patients
Source: Int J Mol Sci. 2022 Sep 7;23(18):10312. doi: 10.3390/ijms231810312 (PMC9499262; doi:10.3390/ijms231810312)
Supplement: Supplementary file 1 [file ijms-23-10312-s001.zip › Supplement Table S1.pdf]

Supplement Table S1. Phagocytic regulatory factor.

Gene	Source or function
GFI1	PMID: 34497417
MUC12	PMID: 34497417
MUC1	PMID: 34497417
LRRC15	PMID: 34497417
MUC21	PMID: 34497417
PODXL	PMID: 34497417
SMAGP	PMID: 34497417
C5AR1	PMID: 34497417
RAC1	PMID: 30397336
DOCK2	PMID: 30397336
NCKAP1L	PMID: 30397336
WASF2	PMID: 30397336
ABI1	PMID: 30397336
CYFIP1	PMID: 30397336
BRK1	PMID: 30397336
ACTR2	PMID: 30397336
ACTR3	PMID: 30397336
ARPC2	PMID: 30397336
ARPC3	PMID: 30397336
ARPC4	PMID: 30397336
RRAGA	PMID: 30397336
LAMTOR2	PMID: 30397336
LAMTOR3	PMID: 30397336
LAMTOR4	PMID: 30397336
NPRL2	PMID: 30397336
NHLRC2	PMID: 30397336
TM2D1	PMID: 30397336
TM2D2	PMID: 30397336
TM2D3	PMID: 30397336
ADGRF5	GOBP_MACROPHAGE_ACTIVATION
AGER	GOBP_MACROPHAGE_ACTIVATION
AIF1	GOBP_MACROPHAGE_ACTIVATION
APP	GOBP_MACROPHAGE_ACTIVATION
ATM	GOBP_MACROPHAGE_ACTIVATION
AZU1	GOBP_MACROPHAGE_ACTIVATION
BPI	GOBP_MACROPHAGE_ACTIVATION
C1QA	GOBP_MACROPHAGE_ACTIVATION
C5AR1	GOBP_MACROPHAGE_ACTIVATION
CCL3	GOBP_MACROPHAGE_ACTIVATION
CD200	GOBP_MACROPHAGE_ACTIVATION
CD74	GOBP_MACROPHAGE_ACTIVATION

CD84	GOBP_MACROPHAGE_ACTIVATION
CD93	GOBP_MACROPHAGE_ACTIVATION
CEBPA	GOBP_MACROPHAGE_ACTIVATION
CLU	GOBP_MACROPHAGE_ACTIVATION
CRTC3	GOBP_MACROPHAGE_ACTIVATION
CSF2	GOBP_MACROPHAGE_ACTIVATION
CST7	GOBP_MACROPHAGE_ACTIVATION
CTSC	GOBP_MACROPHAGE_ACTIVATION
CX3CL1	GOBP_MACROPHAGE_ACTIVATION
CX3CR1	GOBP_MACROPHAGE_ACTIVATION
DYSF	GOBP_MACROPHAGE_ACTIVATION
EDN2	GOBP_MACROPHAGE_ACTIVATION
FCGR2B	GOBP_MACROPHAGE_ACTIVATION
FER1L5	GOBP_MACROPHAGE_ACTIVATION
FOXP1	GOBP_MACROPHAGE_ACTIVATION
FPR2	GOBP_MACROPHAGE_ACTIVATION
GPR137B	GOBP_MACROPHAGE_ACTIVATION
GRN	GOBP_MACROPHAGE_ACTIVATION
HAMP	GOBP_MACROPHAGE_ACTIVATION
HAVCR2	GOBP_MACROPHAGE_ACTIVATION
HSPD1	GOBP_MACROPHAGE_ACTIVATION
IFI35	GOBP_MACROPHAGE_ACTIVATION
IFNG	GOBP_MACROPHAGE_ACTIVATION
IFNGR1	GOBP_MACROPHAGE_ACTIVATION
IFNGR2	GOBP_MACROPHAGE_ACTIVATION
IL10	GOBP_MACROPHAGE_ACTIVATION
IL13	GOBP_MACROPHAGE_ACTIVATION
IL1RL1	GOBP_MACROPHAGE_ACTIVATION
IL31RA	GOBP_MACROPHAGE_ACTIVATION
IL33	GOBP_MACROPHAGE_ACTIVATION
IL4	GOBP_MACROPHAGE_ACTIVATION
IL4R	GOBP_MACROPHAGE_ACTIVATION
IL6	GOBP_MACROPHAGE_ACTIVATION
ITGAM	GOBP_MACROPHAGE_ACTIVATION
ITGB2	GOBP_MACROPHAGE_ACTIVATION
JAK2	GOBP_MACROPHAGE_ACTIVATION
JMJD6	GOBP_MACROPHAGE_ACTIVATION
JUN	GOBP_MACROPHAGE_ACTIVATION
JUND	GOBP_MACROPHAGE_ACTIVATION
KARS1	GOBP_MACROPHAGE_ACTIVATION
LBP	GOBP_MACROPHAGE_ACTIVATION
LDLR	GOBP_MACROPHAGE_ACTIVATION
LRFN5	GOBP_MACROPHAGE_ACTIVATION
LRRK2	GOBP_MACROPHAGE_ACTIVATION

MAPT	GOBP_MACROPHAGE_ACTIVATION
MFHAS1	GOBP_MACROPHAGE_ACTIVATION
MIF	GOBP_MACROPHAGE_ACTIVATION
MIR128-1	GOBP_MACROPHAGE_ACTIVATION
MIR130A	GOBP_MACROPHAGE_ACTIVATION
MIR142	GOBP_MACROPHAGE_ACTIVATION
MIR145	GOBP_MACROPHAGE_ACTIVATION
MIR181C	GOBP_MACROPHAGE_ACTIVATION
MMP8	GOBP_MACROPHAGE_ACTIVATION
MYO18A	GOBP_MACROPHAGE_ACTIVATION
NMI	GOBP_MACROPHAGE_ACTIVATION
NR1D1	GOBP_MACROPHAGE_ACTIVATION
NR1H3	GOBP_MACROPHAGE_ACTIVATION
PJA2	GOBP_MACROPHAGE_ACTIVATION
PLA2G10	GOBP_MACROPHAGE_ACTIVATION
PLA2G3	GOBP_MACROPHAGE_ACTIVATION
PLA2G4A	GOBP_MACROPHAGE_ACTIVATION
PLCG2	GOBP_MACROPHAGE_ACTIVATION
PRKCE	GOBP_MACROPHAGE_ACTIVATION
PTPRC	GOBP_MACROPHAGE_ACTIVATION
RORA	GOBP_MACROPHAGE_ACTIVATION
SBNO2	GOBP_MACROPHAGE_ACTIVATION
SHPK	GOBP_MACROPHAGE_ACTIVATION
SLC11A1	GOBP_MACROPHAGE_ACTIVATION
SNCA	GOBP_MACROPHAGE_ACTIVATION
SPACA3	GOBP_MACROPHAGE_ACTIVATION
SPHK1	GOBP_MACROPHAGE_ACTIVATION
STAP1	GOBP_MACROPHAGE_ACTIVATION
SUCNR1	GOBP_MACROPHAGE_ACTIVATION
SYK	GOBP_MACROPHAGE_ACTIVATION
SYT11	GOBP_MACROPHAGE_ACTIVATION
TAFA3	GOBP_MACROPHAGE_ACTIVATION
THBS1	GOBP_MACROPHAGE_ACTIVATION
TICAM1	GOBP_MACROPHAGE_ACTIVATION
TLR1	GOBP_MACROPHAGE_ACTIVATION
TLR2	GOBP_MACROPHAGE_ACTIVATION
TLR3	GOBP_MACROPHAGE_ACTIVATION
TLR4	GOBP_MACROPHAGE_ACTIVATION
TLR6	GOBP_MACROPHAGE_ACTIVATION
TMEM106A	GOBP_MACROPHAGE_ACTIVATION
TNF	GOBP_MACROPHAGE_ACTIVATION
TNIP2	GOBP_MACROPHAGE_ACTIVATION
TREM2	GOBP_MACROPHAGE_ACTIVATION
TREX1	GOBP_MACROPHAGE_ACTIVATION

TRPV1	GOBP_MACROPHAGE_ACTIVATION
TTBK1	GOBP_MACROPHAGE_ACTIVATION
TYROBP	GOBP_MACROPHAGE_ACTIVATION
VSIG4	GOBP_MACROPHAGE_ACTIVATION
WNT5A	GOBP_MACROPHAGE_ACTIVATION
ZC3H12A	GOBP_MACROPHAGE_ACTIVATION
ABCA7	GOBP_REGULATION_OF_PHAGOCYTOSIS
ADIPOQ	GOBP_REGULATION_OF_PHAGOCYTOSIS
AHSG	GOBP_REGULATION_OF_PHAGOCYTOSIS
ALOX15	GOBP_REGULATION_OF_PHAGOCYTOSIS
ANO6	GOBP_REGULATION_OF_PHAGOCYTOSIS
APOA1	GOBP_REGULATION_OF_PHAGOCYTOSIS
APOA2	GOBP_REGULATION_OF_PHAGOCYTOSIS
APPL1	GOBP_REGULATION_OF_PHAGOCYTOSIS
APPL2	GOBP_REGULATION_OF_PHAGOCYTOSIS
ATG3	GOBP_REGULATION_OF_PHAGOCYTOSIS
ATG5	GOBP_REGULATION_OF_PHAGOCYTOSIS
AZU1	GOBP_REGULATION_OF_PHAGOCYTOSIS
BCR	GOBP_REGULATION_OF_PHAGOCYTOSIS
C2	GOBP_REGULATION_OF_PHAGOCYTOSIS
C3	GOBP_REGULATION_OF_PHAGOCYTOSIS
C4A	GOBP_REGULATION_OF_PHAGOCYTOSIS
C4B	GOBP_REGULATION_OF_PHAGOCYTOSIS
CALR	GOBP_REGULATION_OF_PHAGOCYTOSIS
CAMK1D	GOBP_REGULATION_OF_PHAGOCYTOSIS
CCL2	GOBP_REGULATION_OF_PHAGOCYTOSIS
CD300A	GOBP_REGULATION_OF_PHAGOCYTOSIS
CD300LF	GOBP_REGULATION_OF_PHAGOCYTOSIS
CD36	GOBP_REGULATION_OF_PHAGOCYTOSIS
CD47	GOBP_REGULATION_OF_PHAGOCYTOSIS
CLEC7A	GOBP_REGULATION_OF_PHAGOCYTOSIS
CNN2	GOBP_REGULATION_OF_PHAGOCYTOSIS
COLEC10	GOBP_REGULATION_OF_PHAGOCYTOSIS
COLEC11	GOBP_REGULATION_OF_PHAGOCYTOSIS
CSK	GOBP_REGULATION_OF_PHAGOCYTOSIS
CYBA	GOBP_REGULATION_OF_PHAGOCYTOSIS
DNM2	GOBP_REGULATION_OF_PHAGOCYTOSIS
DOCK2	GOBP_REGULATION_OF_PHAGOCYTOSIS
DYSF	GOBP_REGULATION_OF_PHAGOCYTOSIS
F2RL1	GOBP_REGULATION_OF_PHAGOCYTOSIS
FCER1G	GOBP_REGULATION_OF_PHAGOCYTOSIS
FCGR2B	GOBP_REGULATION_OF_PHAGOCYTOSIS
FCN1	GOBP_REGULATION_OF_PHAGOCYTOSIS
FCN2	GOBP_REGULATION_OF_PHAGOCYTOSIS

FCN3	GOBP_REGULATION_OF_PHAGOCYTOSIS
FER1L5	GOBP_REGULATION_OF_PHAGOCYTOSIS
FGR	GOBP_REGULATION_OF_PHAGOCYTOSIS
FPR2	GOBP_REGULATION_OF_PHAGOCYTOSIS
GAS6	GOBP_REGULATION_OF_PHAGOCYTOSIS
GATA2	GOBP_REGULATION_OF_PHAGOCYTOSIS
HCK	GOBP_REGULATION_OF_PHAGOCYTOSIS
HMGB1	GOBP_REGULATION_OF_PHAGOCYTOSIS
IFNG	GOBP_REGULATION_OF_PHAGOCYTOSIS
IL15	GOBP_REGULATION_OF_PHAGOCYTOSIS
IL15RA	GOBP_REGULATION_OF_PHAGOCYTOSIS
IL1B	GOBP_REGULATION_OF_PHAGOCYTOSIS
IL2RB	GOBP_REGULATION_OF_PHAGOCYTOSIS
IL2RG	GOBP_REGULATION_OF_PHAGOCYTOSIS
ITGA2	GOBP_REGULATION_OF_PHAGOCYTOSIS
ITGAV	GOBP_REGULATION_OF_PHAGOCYTOSIS
LMAN2	GOBP_REGULATION_OF_PHAGOCYTOSIS
LYAR	GOBP_REGULATION_OF_PHAGOCYTOSIS
MBL2	GOBP_REGULATION_OF_PHAGOCYTOSIS
MERTK	GOBP_REGULATION_OF_PHAGOCYTOSIS
MFGE8	GOBP_REGULATION_OF_PHAGOCYTOSIS
MIR17	GOBP_REGULATION_OF_PHAGOCYTOSIS
MIR181B1	GOBP_REGULATION_OF_PHAGOCYTOSIS
MIR183	GOBP_REGULATION_OF_PHAGOCYTOSIS
MIR20A	GOBP_REGULATION_OF_PHAGOCYTOSIS
MYO18A	GOBP_REGULATION_OF_PHAGOCYTOSIS
NCKAP1L	GOBP_REGULATION_OF_PHAGOCYTOSIS
NOD2	GOBP_REGULATION_OF_PHAGOCYTOSIS
OLFM4	GOBP_REGULATION_OF_PHAGOCYTOSIS
PIP4P2	GOBP_REGULATION_OF_PHAGOCYTOSIS
PLA2G5	GOBP_REGULATION_OF_PHAGOCYTOSIS
PLCG2	GOBP_REGULATION_OF_PHAGOCYTOSIS
PLSCR1	GOBP_REGULATION_OF_PHAGOCYTOSIS
PRKCG	GOBP_REGULATION_OF_PHAGOCYTOSIS
PRTN3	GOBP_REGULATION_OF_PHAGOCYTOSIS
PTPRC	GOBP_REGULATION_OF_PHAGOCYTOSIS
PTPRJ	GOBP_REGULATION_OF_PHAGOCYTOSIS
PTX3	GOBP_REGULATION_OF_PHAGOCYTOSIS
PYCARD	GOBP_REGULATION_OF_PHAGOCYTOSIS
RAB27A	GOBP_REGULATION_OF_PHAGOCYTOSIS
RAB31	GOBP_REGULATION_OF_PHAGOCYTOSIS
RACK1	GOBP_REGULATION_OF_PHAGOCYTOSIS
SCARB1	GOBP_REGULATION_OF_PHAGOCYTOSIS
SFTPD	GOBP_REGULATION_OF_PHAGOCYTOSIS

SIRPA	GOBP_REGULATION_OF_PHAGOCYTOSIS
SIRPB1	GOBP_REGULATION_OF_PHAGOCYTOSIS
SIRPG	GOBP_REGULATION_OF_PHAGOCYTOSIS
SLC11A1	GOBP_REGULATION_OF_PHAGOCYTOSIS
SNX3	GOBP_REGULATION_OF_PHAGOCYTOSIS
SOD1	GOBP_REGULATION_OF_PHAGOCYTOSIS
SPACA3	GOBP_REGULATION_OF_PHAGOCYTOSIS
SPHK1	GOBP_REGULATION_OF_PHAGOCYTOSIS
STAP1	GOBP_REGULATION_OF_PHAGOCYTOSIS
SYK	GOBP_REGULATION_OF_PHAGOCYTOSIS
SYT11	GOBP_REGULATION_OF_PHAGOCYTOSIS
SYT7	GOBP_REGULATION_OF_PHAGOCYTOSIS
TGM2	GOBP_REGULATION_OF_PHAGOCYTOSIS
TLR2	GOBP_REGULATION_OF_PHAGOCYTOSIS
TNF	GOBP_REGULATION_OF_PHAGOCYTOSIS
TREM2	GOBP_REGULATION_OF_PHAGOCYTOSIS
TUB	GOBP_REGULATION_OF_PHAGOCYTOSIS
TULP1	GOBP_REGULATION_OF_PHAGOCYTOSIS
