# Supplementary material for: Phagocytosis-Regulators-Based Signature to Predict the Prognosis and Chemotherapy Resistance for Breast Cancer Patients
Source: Int J Mol Sci. 2022 Sep 7;23(18):10312. doi: 10.3390/ijms231810312 (PMC9499262; doi:10.3390/ijms231810312)
Supplement: Supplementary file 1 [file ijms-23-10312-s001.zip › Supplement Table S2.pdf]

Supplement Table S2. Significantly differentially expressed phagocytic factors between normal and tumor samples.

Gene	p_value	pattern
IL33	5.20E-61	N_over_T
CD36	3.30E-59	N_over_T
LAMTOR2	2.50E-54	T_over_N
ADGRF5	1.00E-51	N_over_T
PLA2G4A	1.10E-51	N_over_T
ADIPOQ	2.10E-51	N_over_T
SNCA	1.20E-50	N_over_T
CALR	1.80E-49	T_over_N
LRRC15	1.80E-49	T_over_N
CX3CL1	7.60E-48	N_over_T
LRFN5	1.90E-47	N_over_T
SIRPA	3.30E-46	N_over_T
ARPC3	1.90E-45	T_over_N
ANO6	5.00E-45	N_over_T
IFNGR1	8.10E-45	N_over_T
CD200	9.00E-45	N_over_T
TLR4	3.00E-41	N_over_T
WASF2	8.20E-39	N_over_T
ARPC4	3.80E-38	T_over_N
MFGE8	6.00E-37	N_over_T
PTX3	1.20E-36	N_over_T
JUN	1.70E-36	N_over_T
PLA2G5	2.30E-34	N_over_T
PIP4P2	2.40E-34	N_over_T
MIF	1.10E-33	T_over_N
IL6	2.10E-33	N_over_T
LMAN2	9.40E-33	T_over_N
CSK	9.80E-33	T_over_N
PLCG2	4.30E-32	N_over_T
TLR3	7.10E-31	N_over_T
PYCARD	2.00E-30	T_over_N
OLFM4	2.70E-30	N_over_T
TUB	4.00E-30	N_over_T
MUC1	1.30E-29	T_over_N
SLC11A1	1.70E-28	T_over_N
NR1H3	4.90E-28	N_over_T
RAB31	1.20E-26	T_over_N
MERTK	2.90E-26	N_over_T
C3	3.60E-26	N_over_T
CD93	7.50E-25	N_over_T
CEBPA	3.00E-24	N_over_T

EDN2	7.10E-24	T_over_N
GRN	2.00E-23	T_over_N
LRRK2	2.40E-23	N_over_T
PJA2	1.50E-22	N_over_T
CX3CR1	2.80E-22	N_over_T
NOD2	3.10E-22	T_over_N
CD300LF	2.50E-21	T_over_N
RORA	3.00E-21	N_over_T
PODXL	3.30E-21	N_over_T
CLU	3.70E-21	N_over_T
GAS6	1.80E-20	N_over_T
SOD1	5.50E-20	T_over_N
ACTR2	9.00E-20	T_over_N
IFI35	1.00E-19	T_over_N
IL15RA	4.20E-19	N_over_T
SNX3	5.80E-19	N_over_T
SBNO2	2.60E-18	T_over_N
ACTR3	1.80E-17	T_over_N
TTBK1	2.00E-17	T_over_N
HSPD1	2.90E-17	T_over_N
LAMTOR4	1.30E-16	T_over_N
BPI	2.10E-16	N_over_T
NHLRC2	2.50E-16	N_over_T
DNM2	3.40E-16	T_over_N
COLEC11	4.00E-16	N_over_T
GPR137B	6.20E-16	T_over_N
FCN2	9.60E-16	N_over_T
ARPC2	1.00E-14	T_over_N
FCN3	2.10E-13	N_over_T
APPL2	2.40E-13	N_over_T
JAK2	2.80E-13	N_over_T
ATM	3.00E-13	N_over_T
PLA2G10	6.30E-13	T_over_N
ATG3	6.90E-13	T_over_N
MIR130A	1.60E-12	N_over_T
CLEC7A	3.10E-12	T_over_N
CYBA	3.90E-12	T_over_N
FER1L5	4.00E-12	N_over_T
SCARB1	5.50E-12	N_over_T
VSIG4	6.20E-12	N_over_T
SYT7	1.90E-11	T_over_N
LBP	3.00E-11	N_over_T
TNIP2	3.20E-11	T_over_N
CCL2	6.20E-11	N_over_T

TREM2	8.90E-11	T_over_N
COLEC10	1.50E-10	T_over_N
JUND	1.90E-10	N_over_T
TAFA3	3.20E-10	N_over_T
ITGB2	3.50E-10	T_over_N
NR1D1	3.60E-10	N_over_T
PRKCE	5.70E-10	N_over_T
TYROBP	5.70E-10	T_over_N
SUCNR1	5.80E-10	N_over_T
FCN1	7.80E-10	N_over_T
TM2D2	1.40E-09	T_over_N
APOA1	1.70E-09	T_over_N
PTPRJ	2.30E-09	T_over_N
SIRPG	2.60E-09	T_over_N
TICAM1	3.00E-09	T_over_N
HAVCR2	4.90E-09	T_over_N
TLR1	5.30E-09	N_over_T
IL13	5.70E-09	N_over_T
APP	8.20E-09	N_over_T
IL15	1.70E-08	N_over_T
IL4R	2.60E-08	N_over_T
CD47	4.00E-08	N_over_T
FCER1G	5.60E-08	T_over_N
STAP1	1.10E-07	T_over_N
IL1RL1	1.80E-07	N_over_T
TGM2	2.70E-07	T_over_N
CRTC3	2.90E-07	N_over_T
ITGA2	3.10E-07	N_over_T
C4A	5.50E-07	T_over_N
SYT11	5.90E-07	N_over_T
RAC1	6.30E-07	T_over_N
SFTPD	6.70E-07	N_over_T
C4B	7.80E-07	T_over_N
IL31RA	7.90E-07	T_over_N
RACK1	1.10E-06	N_over_T
C2	1.20E-06	T_over_N
BRK1	2.50E-06	T_over_N
FGR	3.00E-06	N_over_T
NMI	4.20E-06	T_over_N
TLR2	6.80E-06	N_over_T
HAMP	9.10E-06	T_over_N
HCK	1.00E-05	T_over_N
IFNGR2	1.60E-05	T_over_N
CD74	2.90E-05	T_over_N

CD84	4.90E-05	T_over_N
CST7	5.70E-05	T_over_N
MYO18A	6.90E-05	N_over_T
IL2RG	8.60E-05	T_over_N
FOXP1	9.20E-05	N_over_T
IL4	0.00012	N_over_T
RRAGA	0.00014	N_over_T
SPHK1	0.00018	T_over_N
DYSF	0.00037	N_over_T
ABCA7	0.00044	T_over_N
ITGAM	0.00065	T_over_N
MIR181C	0.00073	N_over_T
SYK	0.00073	T_over_N
TULP1	0.00087	N_over_T
RAB27A	0.00095	N_over_T
MMP8	0.002	T_over_N
CYFIP1	0.0022	N_over_T
LDLR	0.0024	N_over_T
MUC12	0.0035	N_over_T
IL1B	0.0059	N_over_T
IL2RB	0.0061	T_over_N
MUC21	0.0069	T_over_N
TMEM106A	0.008	T_over_N
AGER	0.0085	N_over_T
ALOX15	0.0086	N_over_T
BCR	0.0086	N_over_T
CCL3	0.0091	N_over_T
CD300A	0.01	T_over_N
SIRPB1	0.02	T_over_N
ZC3H12A	0.024	N_over_T
IFNG	0.026	T_over_N
MAPT	0.028	T_over_N
F2RL1	0.029	N_over_T
JMJD6	0.031	T_over_N
APPL1	0.032	N_over_T
FCGR2B	0.037	N_over_T
AZU1	0.04	T_over_N
CTSC	0.045	N_over_T

N: normal samples; T: tumor samples.
